# Supplementary material for: Are carbon emissions trading and green financial instruments synergistic? -Comprehensive quantitative research based on content analysis
Source: PLoS One. 2024 Mar 7;19(3):e0298601. doi: 10.1371/journal.pone.0298601 (PMC10919601; doi:10.1371/journal.pone.0298601)
Supplement: S1 Data — (ZIP) [file pone.0298601.s001.zip › Data/地级市Z_F工作报告.zip!/╣π╢1⁄2/╤⌠╜¡╩╨/└┤╫╘╣1⁄2╓┌║┼í╛┬φ┐╦╩2╛▌═°í┐_2014.docx]

各位代表：

　　我代表市人民政府向大会作工作报告，请予审议，并请政协各位委员和其他列席人员提出意见。

　　2013年工作回顾

　　2013年是我市抢抓机遇、铆足干劲，推动经济社会发展取得显著成绩的一年。一年来，在省委、省政府和市委的正确领导下，在市人大及其常委会和市政协的监督支持下，市政府团结带领全市人民，紧紧围绕主题主线和“在加快发展中转变方式，实现幸福追赶”的核心任务，深入实施“双化”驱动、蓝色崛起和融入珠三角战略，着力稳增长、调结构、促改革、惠民生，主要经济指标增幅位居全省前列，幸福阳江建设取得新成效，较好地完成了市六届人大三次会议确定的目标任务。

　　——经济实力稳步提升。采取一系列稳增长的措施，促进了国民经济持续快速健康发展。初步测算，全市地区生产总值突破1000亿元，达1040亿元，增长15.3%;人均生产总值达42000元，增长14.7%，在粤东西北地区率先超过全国同期平均水平;地方公共财政预算收入突破50亿元，达53.7亿元，增长24.5%;规模以上工业总产值1540亿元，增长30%;规模以上工业增加值380亿元，增长31%;固定资产投资595亿元，增长23%;社会消费品零售总额525亿元，增长12.5%;外贸进出口总额23.8亿美元，增长7.1%;实际利用外资1.65亿美元，增长8%;金融机构人民币存款余额813.03亿元、贷款余额524.77亿元，分别增长12.1%和21.9%;城镇居民人均可支配收入21701元，增长13.5%;农村居民人均纯收入10670元，增长16%。

　　——转型升级步伐加快。三次产业比重由19.8:46.1:34.1调整为18.6:49.2:32.2。新型工业化加快推进。重工业总产值增长33.4%，比轻工业快7.8个百分点。工业用电量50.69亿千瓦时，增长33.4%。传统产业改造升级加快，五金刀剪产业、食品(水产品)加工产业转型升级基地建设扎实推进，五金刀剪产业总产值397亿元，增长33.5%。园区经济发展壮大，4个省级产业转移工业园规模以上工业总产值616亿元，增长34.5%，占全市规模以上工业总产值的40%。市政府和广州(阳江)、中山火炬(阳西)产业转移工业园在全省考核中被评为优秀等次。培育主营业务收入超100亿元企业1家。农业结构调整稳步推进。全市农业总产值312亿元，增长4.5%，其中渔业总产值131亿元，增长5.3%。粮食总产量69.8万吨。新建市级农业园区10个，阳江豆(姜)豉、阳江黄鬃鹅获评国家地理标志保护产品。远洋渔业加快发展，深水网箱养殖规模持续扩大，东平渔港升级改造顺利完成，全市水产品总产量115万吨，增长5.5%。第三产业发展势头良好。广东海上丝绸之路博物馆被评为国家4A景区、国家二级博物馆，“南海Ⅰ号”全面保护发掘正式启动。全市接待游客1255.3万人次，旅游总收入114.2亿元，分别增长15.6%和32%。房地产业健康发展，商品房销售面积256万平方米，增长35%;销售额116亿元，增长31%。自主创新能力不断增强。国家刀剪质检中心获国家质检总局批准正式成立，新增省级技术创新专业镇1个、省级工程技术研究开发中心4家、省级名牌产品16个，阳江市和阳东县、阳西县分别荣获全国科技进步考核先进市、先进县。

　　——项目建设成效明显。全市60个重点项目完成投资232.75亿元，增长19%，占全年投资任务的116%。阳江核电站1号机组和华厦阳西电厂3、4号机组并网发电，世纪青山镍合金精炼扩产、阳春海螺水泥一期、阳西博德陶瓷第一二条生产线等6个项目建成投产，厨邦食品等23个续建项目加快建设，阳江核电站5、6号机组和云阳高速公路罗阳段等项目顺利开工。海陵岛大桥、阳江港大桥等项目前期工作有序开展。

　　——招商引资创新突破。创新招商引资方式，推进资源招商和产业招商，策划和实施十大专业招商方案，新引进翌川镍合金、山河游艇等内外资项目104个，总投资290.4亿元。其中，投资额超1亿元项目43个、超5亿元项目15个、超10亿元项目9个。强化引进项目跟踪服务，促进项目顺利实施，形成“投产一批、在建一批、开工一批、储备一批”的发展格局。对外交流取得新进展，阳江与新西兰纳尔逊市建立国际友好城市关系。

　　——城乡面貌持续改善。全市城镇化率达50%，比上年提高了2个百分点。市区扩容提质加快，阳江滨海新区发展总体规划经省政府常务会议审议通过，阳东撤县设区前期工作扎实有效，市区新江东路、金郊路等市政工程完成，马南河综合整治与市区雨污分流管网改造完工，市区排后村等“三旧”改造项目加快实施，城市管理不断加强，住建部拟命名我市为国家园林城市。中心镇建设扎实推进，宜居城乡创建工作取得新成效，4个镇、7条村分别获评省第二批宜居示范城镇、示范村。城乡基础设施进一步完善，三山至核电公路改造等一批交通项目建成，农村公路硬底化建设任务超额完成，21宗重点小(二)型水库除险加固完工，海堤加固达标工程建设顺利，500千伏蝶岭变电站扩建工程建成投产。生态环境建设进一步加强，完成造林9.4万亩，建设生态景观林带202公里、碳汇林3.7万亩;连续14年实现耕地占补平衡;单位生产总值能耗控制在省下达指标范围内，主要污染物总量减排考核连续两年居粤东西北地区首位，连续9年在全省环境保护责任考核中获得优良以上等次。

　　——各项改革深入推进。综合改革成效明显。行政体制改革不断深化，市级卫生计生、食品药品机构改革和第五轮市、县行政审批制度改革基本完成，海陵岛试验区、阳江高新区和阳江日报社、阳江广播电视台干部人事制度改革顺利完成，综合行政执法改革试点工作稳步推进。“两建”工作全面铺开，在全省非试点地区率先实施商事登记制度改革，组建了市12345投诉举报平台。建成了省网上办事大厅阳江分厅和县(市、区)分厅，市、县(市、区)、镇(街道)、村(社区)四级联动一站式代办政务服务体系进一步完善。农村综合改革扎实推进，积极培育发展新型农业经营组织，新增农民合作社460家、省级农业龙头企业6家。集体经济年收入20万元以上行政村政经分离改革全部完成。社会组织改革发展扎实推进，新增社会组织261个。在全省率先开展红十字会体制机制改革。事业单位深化改革进展顺利，组建了市检测检验中心、市工程造价站。新一轮国企改革有序开展，组建了市城投公司、市交投集团、市水务集团和市漠阳置业集团。财税改革取得新进展。

　　——民生事业全面进步。建设幸福广东客观指标居粤东西北地区首位。省、市十件民生实事顺利实施。就业形势保持稳定，新增转移农村劳动力5.47万人，城镇新增就业4.46万人，城镇登记失业率为2.25%。教育事业加快发展，完成了18个教育强镇创建任务，建成启用了两阳中学新校区，组建了市第三中学，整合了市第一职业技术学校与市高级技工学校。文体事业蓬勃发展，冼夫人冯盎将军文化公园建成开放，完成了广播电视“渔船通”、文化信息资源共享等一批文体惠民工程，提前两年实现乡镇农民体育健身工程全覆盖，举办了2013南国书香节暨第二届阳江书展等活动，37个文化馆、图书馆等馆(站)实现免费对外开放，阳春独石仔洞穴遗址列入全国重点文物保护单位。医疗卫生工作得到加强，市人民医院新住院大楼、市妇幼保健院新院动工建设，县级公立医院试点改革稳妥推进，镇村卫生服务一体化管理实现全覆盖。人口计生惠民政策有效落实，免费孕前优生健康检查全面实施，妇女“两癌”免费筛查工作在全省率先开展，低生育水平保持稳定。社会保障体系进一步完善，参加城乡居民社会养老保险103.86万人、医疗保险224.6万人。城乡低保标准和补助水平全部达标，五保供养标准实现了不低于当地上年度农村居民人均纯收入60%的目标。市养老中心新颐养楼竣工，市残疾人康复中心建成启用。新增平价商店59家。新开工建设公租房1333套，建成978套。新一轮扶贫开发“双到”工作全面开展，3340户农村低收入住房困难户住房改造建设顺利完成。抗灾复产成效明显，成功防御强台风“尤特”，实现了“大灾无大难、人员零伤亡”，746户“全倒户”新居在今年元旦前全部建成。市人防“101”工程动工建设。军地军民更加团结。民族宗教、外事侨务、审计、统计、供销、档案、打私、气象、地震等工作取得新成绩。妇女儿童、关工委、老促会、老龄等工作取得新进步。

　　——发展环境不断优化。民主法治建设扎实推进。自觉接受人大、政协的监督，定期向市人大、市政协报告和通报工作情况，办好人大代表建议和政协委员提案。广泛听取各民主党派、工商联、无党派人士及工青妇等群众团体的意见和建议。综治信访维稳工作继续加强。各县(市、区)网上信访分厅建成使用，群众利益诉求渠道进一步畅通;社会治安打防控体系不断健全，全市治安稳中向好，成为全省最少发生抢劫和盗窃现象的3个地级市之一。安全生产形势保持稳定。食品药品安全监管进一步加强。应急管理水平有效提升。“六五”普法深入推进，市政府常务会议学法制度有效落实。党风廉政建设取得新成效，惩治和预防腐败体系不断健全。效能提升年活动深入开展，“庸、懒、散、奢”等不良风气得到有效整治，机关行政效能明显提升。工业企业集中服务月活动扎实开展，帮助企业解决实际困难和问题256宗。

　　各位代表，过去一年的成绩来之不易，这是市委精心谋划、正确领导的结果，是市人大和市政协监督支持、共同努力的结果，是全市人民齐心协力、团结奋斗的结果。在此，我代表市人民政府，向全市人民以及所有关心支持阳江改革发展的同志们、朋友们，表示衷心的感谢!

　　与此同时，我们也清醒地认识到，我市经济社会发展还存在不少困难和问题。主要是：重大项目和大型骨干企业不多，经济持续快速增长动力不足;经济发展方式仍然比较粗放，节能减排和环境保护压力增大;城镇化水平较低，基础设施建设滞后，中心城区辐射带动能力不强;基本公共服务均等化水平不高，改善民生任务仍然艰巨，创新社会管理有待加强;一些部门办事效率不高，作风建设存在薄弱环节。我们将采取措施认真解决。

　　2014年工作安排

　　2014年是全面贯彻落实党的十八届三中全会精神、全面深化改革的开局之年，也是深入实施进一步促进粤东西北地区振兴发展决定的重要一年。市政府工作的总体思路是：深入贯彻落实党的十八届三中全会、中央经济工作会议、习近平总书记系列重要讲话特别是视察广东重要讲话和省委十一届三次全会精神，按照市委六届六次全会的部署要求，紧紧围绕主题主线和“在加快发展中转变方式，实现幸福追赶”的核心任务，坚持稳中求进工作总基调，把改革创新贯穿于经济社会发展各个领域各个环节，大力实施“双化”驱动、蓝色崛起和融入珠三角战略，突出交通基础设施建设、产业园区扩能增效、中心城区扩容提质三大抓手，加快推进产业转型升级，全面统筹城乡发展，切实保障和改善民生，努力开创经济社会发展新局面。

　　全年主要预期目标是：地区生产总值增长12.5%，规模以上工业总产值增长21%，地方公共财政预算收入增长16.5%，固定资产投资增长16%，社会消费品零售总额增长12%，外贸进出口总额增长3%，城镇登记失业率控制在3%以内，城乡居民人均收入增长与经济增长基本同步，居民消费价格涨幅控制在全国平均水平以下，单位生产总值能耗控制在省下达指标范围内。重点抓好八个方面工作：

　　一、促进经济持续快速发展

　　抓好重大项目建设。今年计划安排重点建设项目77个，总投资2167亿元，年度投资228亿元。安排预备重点项目58个，总投资1421亿元。加大在建项目推进力度，确保翌川镍合金等项目投产，加快阳江核电站2—6号机组等项目建设，推动地球卫士环保新材料等项目尽快开工，做好三一工程机械生产基地等项目建设前期工作。加强要素保障，着力破解土地、资金、交通、水电等瓶颈制约，加快项目落地建设。落实项目建设责任制，加大协调、服务和监管力度，确保工程进度和质量。

　　提高招商引资实效。完善招商引资激励机制。实施“1+10”招商计划，大力推进园区招商，扎实开展镍合金产业链、硅基功能材料、金融服务业等十大领域专业招商，强化招商选资，着力引进一批有资金实力、有品牌、有团队的大企业。深入实施“乡贤反哺”工程，引导更多外出企业家回乡投资置业。有针对性地举办招商活动，主动登门拜访客商，邀请客商来阳江考察、投资，进一步提高招商引资的精准率、成功率。

　　促进外贸稳定增长。落实外贸扶持政策，鼓励企业扩大外贸进出口。推进餐厨用品、水产品外贸转型升级示范基地建设，抓好重点企业、重点产品出口工作。组织企业参加各类经贸活动，办好第十三届刀博会。巩固发展美国、欧盟等传统市场，积极开拓南美、东盟、俄罗斯等新兴市场。鼓励有实力的企业到境外投资办厂。加强“大通关”建设，促进贸易便利化。

　　支持民营经济发展壮大。认真落实扶持民营经济发展各项政策措施，为民营企业创造公平竞争的政策环境、市场环境和社会环境。引导支持民资进入基础产业、基础设施、市政公用事业等领域。鼓励金融创新，完善民间融资平台，解决民营企业融资难题。加强服务体系建设，为民营企业提供人力资源、技术研发、法律咨询等综合服务，促进民营经济发展上水平。

　　二、推动产业转型升级

　　促进产业园区扩能增效。推进珠海(阳江)产业转移工业园建设，加强与珠海市合作共建，完善园区管理机构，探索建立利益分享机制，加快园区开发步伐，打造全省经济合作示范区。推动各县(市、区)与珠海所辖县区对接合作，力争各县(市、区)省级产业转移工业园合作共建取得新突破。加大市县共建产业园力度，每个县(市、区)发展2—3个主导产业，带动形成区域产业带和产业集群。盘活存量土地和闲置厂房，提高园区土地使用效益。

　　做大做强工业。抓紧编制沿海临港工业发展规划，引导产业集聚发展，提高产业层次。以广青镍合金等企业为龙头，完善上下游产业链，建设全国高端不锈钢生产基地。大力发展新能源、新材料、生物医药、游艇制造等新兴产业，加快构建现代沿海临港工业体系。推进五金刀剪产业、食品(水产品)加工产业转型升级基地建设，利用高新技术、先进适用技术和信息化改造提升传统产业。培育发展大型骨干企业，力争新增主营业务收入超10亿元企业2家、超50亿元企业1家。鼓励支持个体工商户转型升级为企业，推动规模以下工业企业发展为规模以上工业企业。

　　加快发展服务业。完善旅游基础设施建设，改善旅游市场环境，提高旅游吸引力。推进海陵岛旅游综合开发，规划建设“南海Ⅰ号”文化景区，积极发展海上休闲娱乐等旅游项目，争创国家5A旅游景区。加快建设东平、沙扒、春湾等特色旅游风情小镇。优化商业网点规划布局，抓好城市商业综合体建设，引进大型商贸流通企业，发展现代物流、电子商务等新业态，构建粤西商贸物流中心。推进万村千乡市场工程升级改造和农超对接，促进农村消费。大力发展信息服务、工业设计等生产性服务业，加快发展文化创意等新兴服务业。合理引导住房消费，改善住房供应结构，促进房地产业健康发展。

　　提升农业现代化水平。在稳定粮食生产的基础上，大力发展特色效益农业，着力建设阳东县省级现代农业示范区，扶持省级“菜篮子”基地建设，新建10个市级农业园区，加快发展有机食品、绿色食品和无公害农产品。推进市农产品交易物流中心建设。开展土地承包经营权确权登记，加强土地流转服务，积极培育发展农业龙头企业、农民合作社、专业大户和家庭农场，推进农业产业化经营。完善农业科技、信息服务体系，发展政策性农业保险，加强农产品质量安全监管。

　　加快海洋经济强市建设。强化科学用海意识，加强海洋资源的管控和保护，有序开发海湾、海滨和海岛资源。加快高新区港口片区和阳江海洋经济特色产业基地建设。拓展远洋捕捞业，推动青洲岛、南鹏岛等深水网箱产业园区建设，创建一批水产健康养殖示范场。着力发展海产品精深加工，推进海产品仓储物流设施建设，提高海洋渔业经济效益。完善涉海基础设施，加强港口码头建设，推进对岸、溪头渔港升级改造。综合清理整治阳江港非法养殖设施，加快建设海陵岛国家海洋公园，推进海洋牧场示范区建设，打造省级海洋生态文明示范区。
